# A large inflammatory myofibroblastic tumor involving both stomach and spleen: A case report and review of the literature

**DOI:** 10.3892/ol.2014.2761

**Published:** 2014-12-03

**Authors:** WEN-CHAO CHEN, ZHEN-YU JIANG, FAN ZHOU, ZHENG-RONG WU, GUI-XING JIANG, BU-YI ZHANG, LI-PING CAO

**Affiliations:** 1 Department of General Surgery, The Second Affiliated Hospital, Zhejiang University School of Medicine, Hangzhou, Zhejiang 310009, P.R. China; 2 Department of General Surgery, Qing Chun Hospital, Zhejiang University School of Medicine, Hangzhou, Zhejiang 310009, P.R. China; 3 Department of Pathology, The Second Affiliated Hospital, Zhejiang University School of Medicine, Hangzhou, Zhejiang 310009, P.R. China

**Keywords:** inflammatory myofibroblastic tumor, stomach, spleen, gastrointestinal stromal tumor

## Abstract

Inflammatory myofibroblastic tumor (IMT) is a rare, benign neoplasm that most commonly occurs in pediatric patients; it has been described as a pseudosarcomatous proliferation of spindled myofibroblasts mixed with lymphoplasmacytic cells. IMT has been reported in a number of locations throughout the body; however, cases occurring in the gastrointestinal tract are rare and to date, no case involving both the stomach and spleen has been reported. The current study presents a case of an extremely large IMT invading both the stomach and spleen in a 50-year-old female, presenting with a three-month history of left-sided abdominal distension without abdominal pain, fever or vomiting. As the tumor had invaded the stomach and spleen, it was completely excised and concomitantly, the entire stomach and spleen were removed. Histological examination of the biopsy revealed fascicles of spindle cells in a mixed inflammatory background, with inflammatory cells that were immunopositive for vimentin, smooth muscle actin, and negative for anaplastic lymphoma kinase and CD30, confirming the diagnosis of IMT. Four months following local excision of the mass, accompanied by a total gastrectomy and splenectomy, no abdominal distension, abdominal pain, fever or vomiting were observed and no IMT recurrence was identified.

## Introduction

Inflammatory myofibroblastic tumor (IMT) is an uncommon neoplasm initially described in the lungs by Brunn (1) and was originally denoted as inflammatory pseudotumor until the World Health Organization officially named it IMT. In addition to pulmonary, there are many IMTs that have been described in other organs throughout the body, including the heart, liver and retroperitoneum (2–4). From the literature IMT appears to present most commonly in children and young adults, however, the etiology and pathogenesis of IMT remain ambiguous as infection, surgery, autoimmunity and chromosomal variation have all been hypothesized to contribute to IMT development, however, the exact mechanism remains unclear. Infection, surgery, autoimmunity and chromosomal variation are all hypothesized to contribute to the development of IMT (5). Although it is described as a benign neoplasm, some IMTs have the capacity for distant metastases (6). To the best of our knowledge, there has been no previously reported case of gastric IMT in an adult that invaded the stomach and spleen. The current study reports the case of an extremely large IMT that invaded the stomach and spleen in a 50-year-old female with left-sided abdominal distension for three months. Written informed consent was obtained from the patient.

## Case report

A 50-year-old female presented to the Department of General Surgery, The Second Affiliated Hospital (Hangzhou, China)with a three-month history of left-sided abdominal distension and a lump, without abdominal pain, fever, nausea or vomiting. No symptoms of reflux, melena, hematochezia or change in bowel habits were reported. Urination became more frequent, however, no hematuresis or odynuria were present. On examination, a smooth hard lump with limited mobility was identified in the left hypochondrium. The patient’s medical history was unremarkable and did not include any autoimmune disease or surgery.

Laboratory analyses indicated a microcytic hypochromic anemia with a hemoglobin level of 82 g/l (normal range, 113–151 g/l); hematocrit, 25.9% (normal range, 33.5–45.0%); mean corpuscular volume, 73.3 fl (normal range. 84.0–94.0 fl) and mean corpuscular hemoglobin, 23.1 pg (normal range, 27.0–34.0 pg). The platelet count was 129,000/mm^3^ (normal range, 100,000–300,000/mm^3^). The results for the tumor biological markers including CEA, AFP, CA199, CA125, NSE, SCC and β-hCG were all normal.

**Figure 1:**
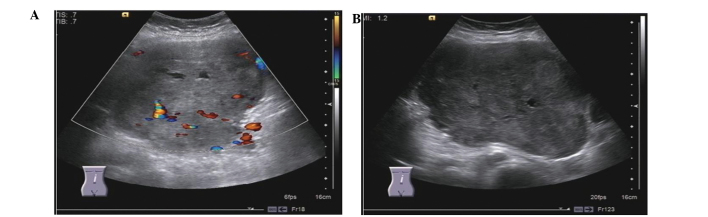
(A) Color Doppler flow imaging revealed some funicular blood flow signals. (B) Abdominal ultrasound showed a large mass with uneven echo and clear edge from the surrounding tissues.

**Figure 2:**
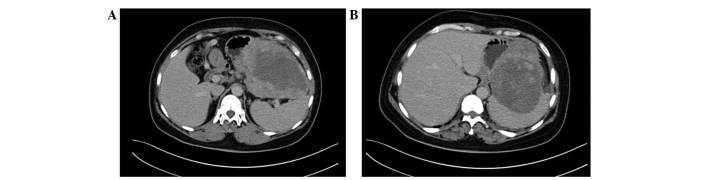
Abdominal computed tomography scan showed the large mass between the greater curvature of stomach and spleen, with (A) a number of enlarged lymph nodes adjacent and (B) invasion of the stomach and spleen.

Ultrasound revealed a mass of 24.5×10.8 cm in size, with its upper margin bound to the edge of liver and its lower margin bound to the umbilical level in the left-sided abdomen (Fig. 1). Abdominal computed tomography revealed a mass of 22×14.2×11 cm in size between the stomach and spleen, causing the surrounding tissues to alter their locations (Fig. 2). Additionally, a number of enlarged retroperitoneal lymph nodes and pelvic effusion were revealed by enhanced computed tomography (Fig. 2).

Exploratory laparotomy showed a large solid mass and numerous varicose vessels were observed on the surface. The mass invaded the greater curvature of stomach and upper spleen with no clear boundary. No liver, omentum or small bowel metastases were identified. Therefore, the patient underwent a wide local excision of the mass, accompanied by a total gastrectomy and splenectomy.

**Figure 3:**
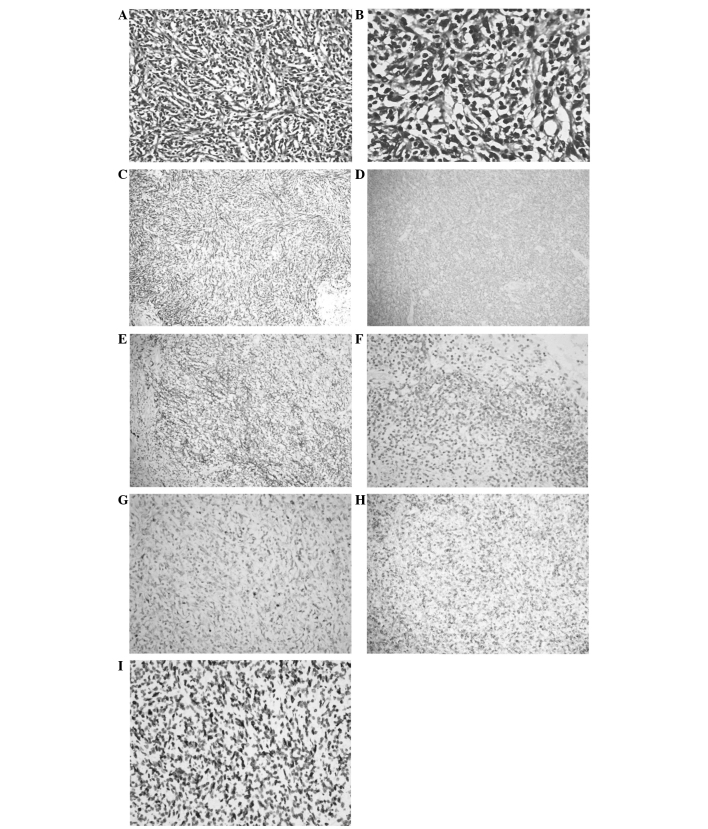
Inflammatory myofibroblastic tumor. Spindle and plump cells in diffuse inflammatory background; hematoxylin and eosin staining for (A) magnification, 100× and (B) magnification, 400x). Immunostaining of (C) vimentin, (D) smooth muscle actin, (E) CD23, (F) CD21, (G) CD68 and (H) LCA. (I) *In situ* hybridization was positive for EBV.

On macroscopic examination, an extremely large mass, measuring 22×13×8.5 cm, was tightly adhered to the stomach and spleen. In the stomach lesion, tumor tissue invaded the entire gastric wall and the overlying mucosa appeared ulcerated (7). Lymph nodes along the greater curvature were excised and underwent biopsy, the result of which was negative for tumor cells. On microscopic examination, a neoplasm composed of spindle cells in inflammatory background with mixed lymphocytes, plasma cells and eosinophils was observed (Fig. 3). Immunohistochemical staining was positive for vimentin, smooth muscle actin (SMA), and negative for CD30 and anaplastic lymphoma kinase (ALK). There were a number of cells expressing CD68, but negative for LCA, excluding the possibility that the tumor was derived from lymphatic and hematopoietic system. Furthermore, for markers of dendritic cell neoplasms, including CD21 and CD35, staining was negative. Additionally, *in situ* hybridization revealed positive results for EBV and EBVR. Based on these results, which were confirmed by the Department of Pathology at the University of California, Los Angeles (UCLA), a diagnosis of IMT was determined. The patient experienced right-sided lower limb venous thrombosis, however, four months following surgery, her recovery is favorable.

## Discussion

IMT (also known as inflammatory pseudotumor, plasma cell granuloma, pseudosarcomatous myofibroblastic lesion, and inflammatory myofibrohistiocytic lesion) was initially described in the lungs (1). Following this, a debate into whether IMT is a tumor or inflammation, and whether it is benign or malignant arose, as it was considered to have the potential of local recurrence and distant metastases; subsequently, the World Health Organization classified IMT as a neoplasm of intermediate biologic potential in 2002 (7,8). IMT is histopathologically composed of myofibroblastic spindle cells infiltrated with inflammatory cells, including plasma cells, lymphocytes and eosinophiles (7). Extrapulmonary IMTs occur in numerous sites throughout the body, including the heart, liver, retroperitoneum, orbit and central nervous system (2–5,9). The first case with abdominal localization was described in the liver by Pack and Baker (10). IMT is most frequently observed in children and those <50 years old, and primary gastric IMT in adults is rare (11).

Although it has been >70 years since the first reported case of lung IMT (1), the etiology of IMT is contentious. It has been proposed to be related to infection, trauma, surgery, autoimmunity and chromosomal variation, such as the abnormalities of ALK gene (12–15). Viruses that were also hypothesized to affect the development of IMTs include HIV, HHV-8 and EBV (16–18). In the present case, while immunohistochemical analyses showed negative staining for ALK, *in situ* hybridization revealed infection of EBV, which may account for the occurrence of IMT. As EBV infection is considered to be related to the neoplastic process of IMTs, a longer follow-up is required for patients with EBV-positive IMTs compared with those with EBV-negative IMTs (17).

Even with a thorough diagnostic workup, distinguishing IMT from other celiac malignancies is challenging. Patients with IMTs most commonly present with symptoms of pain, anemia, fatigue and weight loss, however, these symptoms are not unique to IMTs. Therefore, pathological and immunocytochemical analyses are the ‘gold standards’ for the diagnosis of IMTs. Coffin *et al* reported three histological patterns of IMTs: A myxoid vascular pattern, resembling nodular fasciitis; a compact spindle cell pattern with a fascicular or storiform cellular arrangement; and hypocellular collagenized pattern resembling a scar or desmoid (7). In the present case, histological examination revealed fascicles of spindle cells in a mixed inflammatory background, with plasma cells. Immunocytochemistry showed positive staining for vimentin, SMA, and negative for ALK and CD30. Negative staining was also observed for CD21 and CD35, eliminating the possibility of dendritic cell neoplasm. Coffin *et al* (5) also reported that almost 56% IMT cases exhibited diffuse cytoplasmic ALK expression, however, a number of studies identified IMTs in the spleen and lymph node that showed no ALK overexpression, indicating that the splenic and nodal IMTs may represent a discrete subset that are biologically distinct from other IMTs (5,19,20). In order to further confirm the diagnosis, the clinical information and the results of immunohistochemical analyses were sent to UCLA; the Department of General Surgery (The Second Affiliated Hospital) and UCLA determined a diagnosis of IMT.

To date, the predominant therapy for the treatment of IMTs remains complete resection of the tumor. In addition to surgery, treatment with corticosteroids has also led to tumor regression (21). For cases where IMT cannot be completely resected or in the instance of metastatic disease, chemotherapy is used despite the lack of definitive data for the efficacy. The case presented in the current study showed a rare mass of 22×14.2×11 cm in size that invaded the stomach and spleen; this was marginally smaller than the largest reported IMT described in liver, which measured 25 cm in diameter (22). Therefore, the most favorable treatment option was the complete resection of the tumor with total gastrectomy and splenectomy.

In recent years ALK-directed therapy, a novel treatment option, has been developed, and has already been demonstrated to have a partial therapeutic effect on IMT (23). The ALK fusion gene exists in ~56% of IMTs and has been hypothesized to have significant effects in the process of tumor development (5). A recent phase I trial of crizotinib demonstrated a long-term partial response in a patient with IMT carrying an ALK translocation, but not in a patient with ALK-negative disease (23). However, the emergence of resistance to crizotinib occurred approximately 5–8 months after the initiation of therapy (23). The development of more selective ALK inhibitors, which can overcome emergent crizotinib resistance mutations, will be key to achieving success in the future.

In conclusion, the current study reports the first case of IMT that invaded the stomach and spleen, occurring in a 50 year-old female. In this case, it was important for the tumor to be resected completely, as the volume of tumor was extremely large and the efficacy of other treatment modalities are currently unclear.

## Abbreviations

IMT: Inflammatory myofibroblastic tumor
SMA: smooth muscle actin
ALK: anaplastic lymphoma kinase
